# Changes in Physicochemical and Biological Properties of Polyphenolic-Protein-Polysaccharide Ternary Complexes from *Hovenia dulcis* after In Vitro Simulated Saliva-Gastrointestinal Digestion

**DOI:** 10.3390/foods10102322

**Published:** 2021-09-29

**Authors:** Ding-Tao Wu, Yuan He, Meng-Xi Fu, Ren-You Gan, Yi-Chen Hu, Liang Zou

**Affiliations:** 1 Key Laboratory of Coarse Cereal Processing (Ministry of Agriculture and Rural Affairs), Sichuan Engineering & Technology Research Center of Coarse Cereal Industrialization, School of Food and Biological Engineering, Chengdu University, Chengdu 610106, China; mxfu_1996@163.com (M.-X.F.); ganrenyou@caas.cn (R.-Y.G.); huyichen0323@126.com (Y.-C.H.); 2 Institute of Food Processing and Safety, College of Food Science, Sichuan Agricultural University, Ya’an 625014, China; yhsicau@163.com; 3 Research Center for Plants and Human Health, Institute of Urban Agriculture, Chinese Academy of Agricultural Sciences, Chengdu 610213, China

**Keywords:** *Hovenia dulcis*, polyphenolic-protein-polysaccharide, in vitro digestion, physicochemical properties, biological activities

## Abstract

The present study aimed to explore the impacts of in vitro simulated saliva-gastrointestinal digestion on physicochemical and biological properties of the polyphenolic-protein-polysaccharide ternary complex (PPP) extracted from *Hovenia dulcis*. The results revealed that the in vitro digestion did remarkably affect physicochemical properties of PPP, such as content of reducing sugar release, content of bound polyphenolics, and molecular weight distribution, as well as ratios of compositional monosaccharides and amino acids. In particular, the content of bound polyphenolics notably decreased from 281.93 ± 2.36 to 54.89 ± 0.42 mg GAE/g, which might be the major reason for the reduction of bioactivities of PPP after in vitro digestion. Molecular weight of PPP also remarkably reduced, which might be attributed to the destruction of glycosidic linkages and the disruption of aggregates. Moreover, although biological activities of PPP obviously decreased after in vitro digestion, the digested PPP (PPP-I) also exhibited remarkable in vitro antioxidant and antiglycation activities, as well as in vitro inhibitory effects against α-glucosidase. These findings can help to well understand the digestive behavior of PPP extracted from *H. dulcis*, and provide valuable and scientific supports for the development of PPP in the industrial fields of functional food and medicine.

## 1. Introduction

*Hovenia dulcis* (Rhamnaceae), commonly known as Japanese grape, ‘Guai Zao’ and ‘Zhi Ju’, is native to East Asia such as Japan, Korea, and China [1]. It is a delicious and sweet fruit, and it is also widely used as a functional food and a traditional Chinese herb in China [2]. Generally, *H. dulcis* has a long history been used as a traditional Chinese herb for the treatment of liver disease and alcohol poisoning [1,2]. Recently, a numerous number of in vitro and in vivo studies have demonstrated that *H. dulcis* possesses diverse health-promoting effects, such as anti-diabetic, anti-cancer, anti-oxidant, anti-inflammatory, and hepatoprotective effects [3,4]. Usually, these health-promoting effects are correlated to different bioactive ingredients that exist in *H. dulcis*, such as polyphenolic-protein-polysaccharide ternary complexes, polysaccharides, polyphenolics, flavonoids, and saponins [1,2,3,5,6,7]. Especially, polyphenolic-protein-polysaccharide ternary complexes obtained from *H. dulcis* also possess diverse bioactivities, including anti-oxidant, anti-glycation, and anti-diabetic effects [2,5]. Therefore, all the data cited above indicates that the polyphenolic-protein-polysaccharide ternary complex (PPP) from *H. dulcis* has great potential for the development and application in the healthy food and pharmaceutical industries in terms of strong bioactivities.

Digestion is a multi-stage process for food digestion and absorption in the upper gastrointestinal tract of the human body, including oral cavity, esophagus, stomach, and duodenum [8]. Recently, the simulated saliva-gastrointestinal digestion of active compounds like polysaccharides and proteins has attracted people’s attention due to its effective, convenient, and economical properties [9,10]. For example, researches have indicated that the saliva-gastrointestinal digestion medium such as pH value and bile salts, as well as enzymes can affect chemical properties and biological functions of some polysaccharides, such as polysaccharides extracted from *Plantago asiatica* L. [11] and *Inonotus obliquus* [12]. Besides, Moyo et al. [13] investigated the digestive behavior of phenolic compounds from *Solanum nigrum* complex, finding that phenolic compounds reduced after in vitro digestion but bioactivities still retained. Furthermore, Lorieau et al. [14] utilized in vitro digestion model to figure out the digestive properties of whey protein in different structures. However, there are few reports on the digestive properties of naturally occurring binary complexes and ternary complexes, such as polyphenolic-protein and polyphenolic-protein-polysaccharide (PPP). The PPP extracted from *H. dulcis* is exactly one of the naturally ternary complexes, and the effects of human upper gastrointestinal tract system on chemical properties and biological functions of PPP are still unknown.

Therefore, for the first time, this study aimed to broaden knowledge about the impacts of in vitro digestion on PPP extracted from *H. dulcis*. The changes in physicochemical and biological properties of PPP after in vitro digestion were explored to provide useful information for the future application of *H. dulcis* in food and medicine fields.

## 2. Materials and Methods

### 2.1. Materials and Chemicals

The fresh peduncles of *H. dulcis* were harvested in AnKang City, Shaanxi Province, China.

ABTS (2,2-azidobisphenol (3-ethylbenzthiazoline-6-sulphonic acid)), DPPH (2,2-diphenyl-1-(2,4,6-trinitrophenyl) hydrazyl), sodium nitroprusside (SNP), BSA (bovine serum albumin), α-glucosidase, PMP (1-phenyl-3-methyl-5-pyrazolone), BHT (butylated hydroxytoluene), AG (aminoguanidine), Vc (vitamin C), monosaccharide standards, and amino acid standards were bought from Merck. Analytical grade of other reagents and chemicals were used.

### 2.2. Extraction of Polyphenolic-Protein-Polysaccharide Ternary Complexes

The preparation of the PPP referred to the hot-water extraction method as described in our previous report [5]. Briefly, 10.0 g of *H. dulcis* powders were extracted twice with deionized water (1: 30, *w/v*) at 95 °C for 3 h. After removing starch and protein by thermal stable α-amylase and pancreatin, four volumes of 95% ethanol (*v/v*) were used for precipitation of the supernatant overnight at 4 °C. Then, the precipitate was collected, redissolved in distilled water, and then dialyzed against deionized water (molecular weight cutoff at 3500 Da). Finally, PPP was obtained by freeze drying for further analysis.

### 2.3. In Vitro Simulated Saliva-Gastrointestinal Digestion of PPP

The in vitro digestion of PPP was carried out based on the previous method with slight modifications [15]. The simulated salivary solution (100.0 mL) consisted of KSCN (0.364 g), NaCl (3.187 g), KCl (1.629 g), NaHCO_3_ (1.540 g), Na_2_SO_4_ (1.036 g), NaH_2_PO_4_ (1.615 g), urea (0.455 g), and α-amylase (75 U/mL), and mixed with 100.0 mL of PPP (20.0 mg/mL), then the mixture was incubated at 37 °C for 5 min. Subsequently, 200.0 mL of simulated gastric solution, which composed of 2 g of pepsin, was added into the salivary juice digested sample. The pH of the mixed solution was promptly modified to pH = 2.0 by using HCl, and then incubated at 37 °C for 2 h. Moreover, the pH of the saliva-gastric digested solution was modified to pH = 6.8 during the intestinal digestion, and a mixed solution of pancreatin (3.5 mg/mL) and bile salts (22.0 mg/mL) at a ratio of 3: 10 (*v/v*) was added to the saliva-gastric digested solution. After the intestinal digestion for 2 h, the resulting mixture was boiled in water for 10 min to ensure inactivation of enzymes, and centrifuged to collected supernatant. Then, an aliquot of 2.0 mL sample was taken out for determining the content of reducing sugar release after in vitro digestion of PPP. The remaining part was successively precipitated by ethanol, dialyzed (molecular weight cutoff at 3500 Da), and lyophilized for later use. Finally, the obtained sample was named as PPP-I after the entire in vitro digestion.

### 2.4. Physicochemical Characterization of PPP after In Vitro Digestion

The chemical components, including total polysaccharides, proteins, and uronic acids, as well as bound polyphenolics, were detected according to our formerly reported colorimetric methods [2,5], respectively. Besides, molecular weight distribution was detected by size exclusion chromatography coupled with a multi angle laser light scattering and a refractive index detector (SEC-MALLS-RID, Wyatt Technology Co., Santa Barbara, CA, USA) also referred to the previous study [5], and a Discovery Hybrid Rheometer-1 (TA Instruments, New Castle, DE, USA) was used to detect the apparent viscosities of PPPs referred to the previous study [5]. In addition, the U3000 HPLC system (Thermo Fisher Scientific, Waltham, MA, USA) equipped with a C18 column (150 mm × 4.6 mm, 5 μm) and a diode array detector followed by PMP derivatization was applied for analyzing monosaccharide compositions and reducing sugars [5]. Moreover, the FT-IR analysis was performed by a Nicolet iS 10 FT-IR (Thermo Fisher Scientific, Waltham, MA, USA) referred to the former method [5]. Finally, amino acid compositions were also detected referred to a former method with slight modifications [2]. In brief, 5.0 mL of 6 M HCl was used for the hydrolysis of each sample (20.0 mg) in a sealed tube at 110 °C for 24 h. Then, the degradations were dried by evaporation, and redissolved in 2.0 mL of 0.02 M HCl. Finally, an automated amino acid analyzer (HITACHIL-8900, Amino Acid Analyzer, Tokyo, Japan) was used to analyze amino acid compositions of PPPs.

### 2.5. Evaluation of In Vitro Antioxidant and Antiglycation Effects, as Well as In Vitro Inhibitory Effect against α-Glucosidase of PPP after In Vitro Digestion

Antioxidant capacity indicators, including ABTS, DPPH, and nitric oxide (NO) radical scavenging activities, as well as ferric reducing antioxidant powers (FRAP) of PPP and PPP-I were determined and compared based on a previous study [5]. Briefly, 200 μL of ABTS radical cation solution (absorbance at 734 nm, 0.75) was mixed with 20 μL of PPP and PPP-I at the concentrations ranged from 0.05 mg/mL to 0.25 mg/mL, and from 0.60 mg/mL to 1.00 mg/mL, respectively, and then the mixture was incubated at 30 °C for 20 min. The absorbance was measured at 734 nm. In addition, 200 µL of DPPH solution (0.35 mM) was mixed with 20 µL of PPP and PPP-I at the concentrations ranged from 0.05 mg/mL to 0.25 mg/mL, and from 0.20 mg/mL to 1.00 mg/mL, respectively, and then the mixture was reacted at 37 °C for 30 min. The absorbance at 517 nm was measured. Furthermore, 50 μL of sodium nitroprusside (10 mM) was mixed with 450 μL of PPP and PPP-I at the concentrations ranged from 0.1 mg/mL to 0.5 mg/mL, and from 0.2 mg/mL to 1.0 mg/mL, respectively, and then incubated at 25 °C for 3 h. Then, 250 μL of Griess reagent was added into the mixture. The absorbance was measured at 540 nm. Moreover, 3.0 mL of FRAP solution was mixed with 100 μL of PPP and PPP-I at the concentrations ranged from 0.1 mg/mL to 0.5 mg/mL, and from 1.0 mg/mL to 3.0 mg/mL, respectively. After 4 min of incubation at 37 °C, the absorbance was measured at 593 nm. The positive controls were designated as BHT and vitamin C.

The BSA/glucose model was used to measure the antiglycation activities of PPP and PPP-I referred to a previously reported study [5]. The antiglycation activity of each sample was measured at five concentrations ranged from 0.25 to 4.00 mg/mL. Aminoguanidine (AG) was used as the positive control.

The inhibitory effect of PPP and PPP-I on α-glucosidase was also conducted referred to a reported method [16]. The inhibitory effects against α-glucosidase of PPP and PPP-I were detected at the concentrations ranged from 10 μg/mL to 18 μg/mL, and from 40 μg/mL to 120 μg/mL, respectively. Acarbose was used as the positive control.

### 2.6. Statistical Analysis

Data were expressed as mean ± standard deviation, which were analyzed by using Origin 9.0 software (Origin Lab Corporation, Northampton, MA, USA). Statistical significances (*p* < 0.05) were carried out by one-way analysis of variance (ANOVA) followed by a Duncan’s test.

## 3. Results and Discussion

### 3.1. Effects of In Vitro Digestion on Physicochemical Properties of PPP

#### 3.1.1. Changes in Reducing Sugars, Total Polysaccharides, Total Bound Polyphenolics, and Total Proteins of PPP after In Vitro Digestion

Reducing sugar (CR) content has been used to evaluate the degradation of polysaccharide for a long time and can reveal the breakdown of glycosidic bonds [17]. In this study, the CR content released from PPP raised up to 0.27 ± 0.02 mg/mL after in vitro digestion, indicating that the glycoside bonds were broken in PPP, which might be in connection with the enzymes and low pH value in digestive juices, resulting in the degradation of polysaccharides [8]. Indeed, the major type of reducing sugar was determined as glucose by HPLC analysis. The contents of total polysaccharides and uronic acids in PPP decreased from 43.94% ± 1.52% to 40.33% ± 4.37%, and from 22.41% ± 1.17% to 17.68% ± 0.73%, respectively, after in vitro digestion, which was corresponded to the increase of reducing sugar content in the digestive mixture.

Furthermore, a high content (281.93 ± 2.36 mg GAE/g) of bound polyphenolics was observed in PPP, and thirteen individual phenolic compounds, including protocatechuic acid, gallocatechin, p-hydroxybenzoic acid, ampelopsin, quercetin-7,4′-diglucoside, quercetin, rutin, dihydroquercetin, myricitrin, myricetin, kaempferol, 5-methylmyricetin, and naringenin were identified in PPP [2,5]. Therefore, we investigated the impact of in vitro digestion on the stability of bound polyphenolics in PPP. Results showed that in vitro digestion could also remarkably affect the content of polyphenolic in PPP. As shown in Table 1, after in vitro digestion, the content of total bound polyphenolics remarkably reduced from 281.93 ± 2.36 to 54.89 ± 0.42 mg GAE/g, indicating that bound polyphenolics were released from PPP during in vitro digestion. Similar results were also found in previous studies [18,19,20]. The low pH around 2.0 and the pepsin action in the gastric digestion did have an influence on the stability of bound polyphenolics [19,21]. Additionally, some polyphenolics will be degraded during the transition from the acidic gastric conditions to the mild alkaline intestinal conditions, especially under the influence of bile acids and pancreatin [22].

Moreover, due to the decrease of total polysaccharides and total bound polyphenolics, the proportion of proteins in PPP increased by 27.30% after in vitro digestion. Results suggested that the protein was relatively more stable compared with polysaccharides and bound polyphenolics. Previous studies have shown that phenolic compounds could impact on protein digestibility as they exhibited strong binding ability with proteins [23,24].

#### 3.1.2. Changes in Molecular Weight and Apparent Viscosity of PPP after In Vitro Digestion

**Figure 1:**
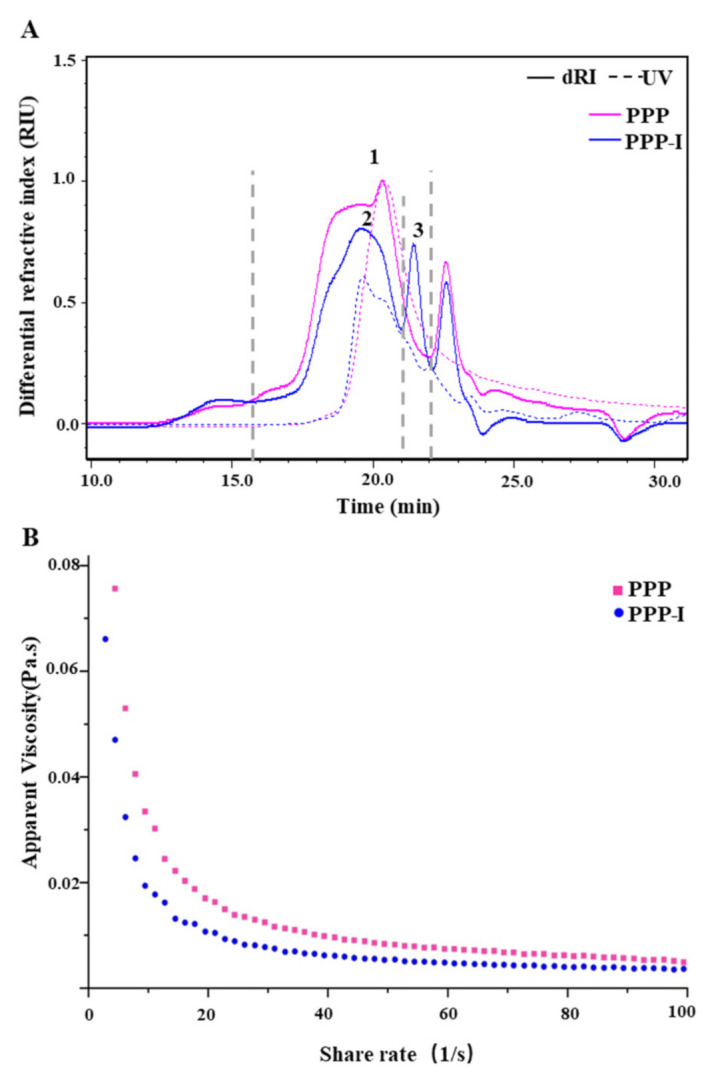
Changes in HPSEC chromatogram (**A**) and apparent viscosity (**B**) of PPP after in vitro digestion. **PPP**, polyphenolic-protein-polysaccharide ternary complexes from *Hovenia dulcis*; **PPP-I**, indicated PPP after in vitro digestion.

In general, the molecular weight (*M_w_*) and apparent viscosity are vital characteristics of natural polysaccharides which can affect their biological activities [5]. Thus, the impacts of in vitro digestion on the *M_w_* and apparent viscosity of PPP were explored. Figure 1A shows the HPSEC-RID-UV chromatograms of PPP and PPP-I. As seen in Table 2, molecular weights of fraction 1 in PPP were detected as 5.97 × 10^4^ Da. However, after in vitro digestion, the molecular weight of PPP changed markedly. It was obvious that the fraction 1 (16 min to 22 min) of PPP degraded into the fraction 2 (16 min to 21 min) and fraction 3 (21 min to 22 min) of PPP-I after in vitro digestion. Moreover, the molecular weights of two fractions in PPP-I remarkably decreased to 4.68 × 10^4^ Da and 1.39 × 10^4^ Da when compared with PPP. The reduction of molecular weight of PPP may be caused by the destruction of glycosidic bonds according to the obvious increase of reducing sugar content after in vitro digestion [25]. It is in accord with former studies, which suggested that natural polysaccharides could be degraded in the digestive juices [26]. In addition, the remarkable decrease of bound polyphenolics might also contribute to the decrease of its molecular weight due to the disruption of aggregates [27].

As shown in Figure 1B, it could be observed that the shear rate made a difference to the apparent viscosities of PPP and PPP-I solution. Both of them possessed shear-thinning and Newtonian fluid behaviors. In addition, a decrease of apparent viscosity of PPP was observed after in vitro digestion. Generally, the apparent viscosity of natural polysaccharides is related to their chemical structure, such as molecular weight distribution and degree of esterification [28]. Therefore, the decrease of apparent viscosity might be corresponded to the reduction of molecular weight of PPP after in vitro saliva-gastrointestinal digestion.

#### 3.1.3. Changes in Monosaccharide Compositions and FT-IR Spectra of PPP after In Vitro Digestion

**Figure 2:**
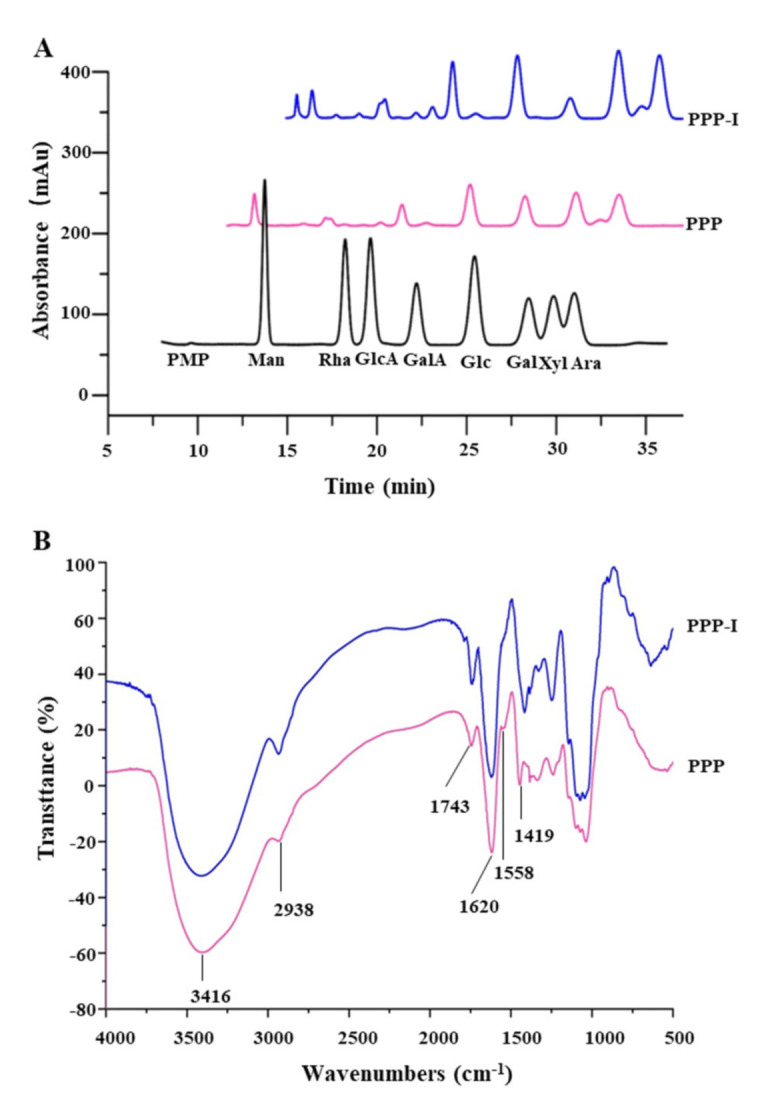
Changes in monosaccharide compositions (**A**) and FT-IR spectra (**B**) of PPP after in vitro digestion. **PPP**, polyphenolic-protein-polysaccharide ternary complexes from *Hovenia dulcis*; **PPP-I**, indicated PPP after in vitro digestion.

It is universally acknowledged that biological functions of natural polysaccharides are also affected by their monosaccharide compositions and molar ratios [27]. Therefore, we also investigated the monosaccharide composition of PPP after in vitro digestion. Figure 2A presents the chromatography profiles of monosaccharides in PPP and PPP-I. The results demonstrated that the monosaccharides in PPP and PPP-I were similar, which mainly consisted of galacturonic acid (GalA), galactose (Gal), arabinose (Ara), glucose (Glc), rhamnose (Rha), mannose (Man), glucuronic acid (GlcA), and xylose (Xyl). Furthermore, GalA, Gal, Ara, Glc, and Rha were determined as the dominant monosaccharides, which were consistent with a previous study [5]. However, their molar ratios had some changes. For instance, after in vitro digestion, the molar ratio of GalA reduced from 1.78 to 1.30, which corresponded to an obvious reduction in total uronic acids (Table 1). Additionally, the molar ratio of Glc also decreased from 0.96 to 0.34. To sum up, results suggested that the types of monosaccharides of PPP were not affected by the saliva-gastrointestinal digestion, but the molar ratios were changed after in vitro digestion.

The primary functional groups of PPP and PPP-I were further evaluated by FT-IR spectroscopy. Figure 2B showed that the FT-IR spectra of PPP and PPP-I were similar, which meant the major functional groups of PPP had no remarkable changes after the saliva-gastrointestinal digestion. Specifically, the strong absorption band around 3416 cm^−1^ could be attributed to the stretching vibrations of -OH, while the relatively band peak at approximately 2938 cm^−1^ was the result of C-H vibration [7]. The band at 1743 cm^−1^ was attributed to be stretching vibration of carboxylic ester (C=O) [5]. Besides, the band that appeared around 1620 cm^−1^ was due to the stretching vibration of C=O asymmetric stretching of ionic carboxyl groups (COO-), suggesting the presence of uronic acids in PPP and PPP-I [1]. The absorption bands at 1620 cm^−1^ and 1558 cm^−1^ that recognized as the amide I and amide II regions were also observed [5]. Furthermore, the band at 1419 cm^−1^ was attributed to C=C stretching vibrations of aromatic ring [2,5].

#### 3.1.4. Changes in Amino Acid Compositions of PPP after In Vitro Digestion

As presented in Table 3, the protein fraction of PPP and PPP-I contained a total of 17 types of amino acids. The results indicated that the amino acid compositions in PPP and PPP-I were similar, and glutamic acid, aspartic acid, proline, serine, glycine, threonine, leucine, and alanine were determined as predominant amino acids in both PPP and PPP-I. However, contents of several amino acids in PPP obviously changed after in vitro digestion. For instance, the ratio of aspartic acid increased from 9.65% to 13.28% after in vitro digestion, which could be used as a carrier of potassium and magnesium ions to improve the myocardial systolic function and protect the myocardium [29]. Similarly, the ratio of lysine increased greatly as well, which could enhance the immune system, and promote bone and prevent osteoporosis combined with other amino acids [30]. Commonly, essential amino acids cannot be synthesized in the human body and must be obtained from daily food. Thus, the quality of protein usually depends on the content of essential amino acids [31]. However, the ratio of essential amino acids decreased from 41.59% to 36.49% after in vitro saliva-gastrointestinal digestion, indicating that some essential amino acids might be destroyed by the acid hydrolysis catalyzed by pepsin during in vitro digestion.

### 3.2. Effects of In Vitro Simulated Saliva-Gastrointestinal Digestion on Biological Functions of PPP

#### 3.2.1. Stability of Antioxidant Activities of PPP after In Vitro Digestion

**Figure 3:**
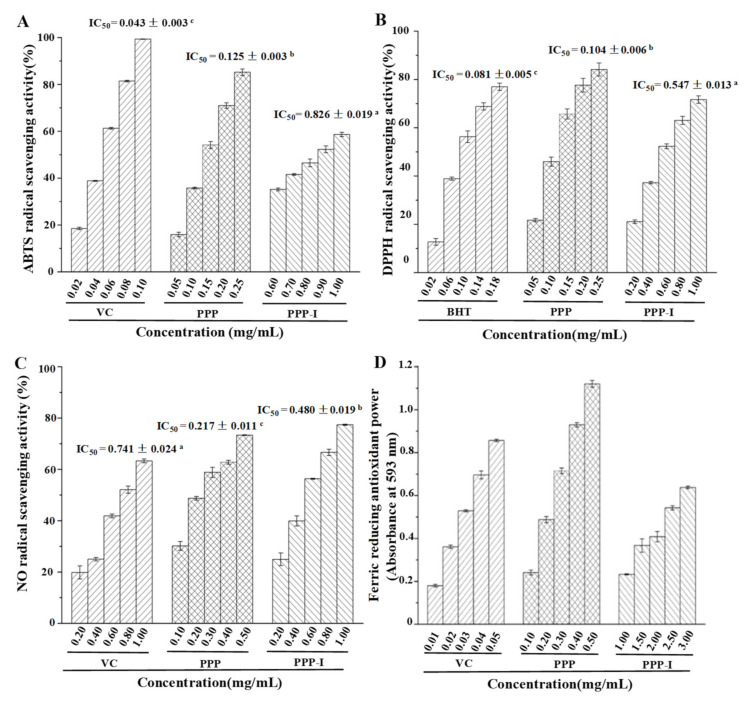
Stability of ABTS (**A**), DPPH (**B**), NO (**C**), and FRAP (**D**) antioxidant capacities of PPP after in vitro digestion. **PPP**, polyphenolic-protein-polysaccharide ternary complexes from *Hovenia dulcis*;** PPP-I**, indicated PPP after in vitro digestion. **BHT**, butylated hydroxytoluene; **Vc**, vitamin C. The error bars are standard deviations; Different superscript lowercase letters (**a**–**c**) indicated significance (*p* < 0.05). Statistical significances (*p* < 0.05) were carried out by one-way analysis of variance (ANOVA) followed by a Duncan’s test.

Previous studies have found that polyphenolic-protein-polysaccharide ternary complexes from *H. dulcis* possessed high antioxidant capacity [2,5]. Hence, the effect of in vitro digestion on the antioxidant ability of PPP was evaluated by different antioxidant assays in the present study, namely ABTS, DPPH, and NO radical scavenging activities, as well as ferric reducing antioxidant powers (FRAP). Figure 3 shows the stability of ABTS, DPPH, and NO radical scavenging activities, as well as FRAP of PPP after in vitro digestion. Compared with the positive controls, experimental results indicated that PPP and PPP-I possessed remarkable antioxidant abilities. The IC_50_ values of ABTS, DPPH, and NO radical scavenging activities of PPP and PPP-I were calculated and ranged from 0.125 to 0.826 mg/mL, from 0.104 to 0.547 mg/mL, and from 0.217 to 0.480 mg/mL, respectively. After in vitro digestion, the antioxidant activities of PPP have reduced sharply (Figure 3), suggesting that the antioxidant activities of PPP were quite unstable during in vitro digestion. The decrease of antioxidant activities of PPP might be corresponded to the changes of its physicochemical properties during in vitro digestion. Obviously, the FRAP of PPP-I was also obviously lower than that of PPP (Figure 3D).

Generally, chemical structures, *M_w_*, and monosaccharide compositions especially uronic acids of polysaccharides can affect their in vitro antioxidant activities [32]. Indeed, for the polyphenolic-protein-polysaccharide complexes, their antioxidant activities are also closely related to polyphenolics and proteins [2,5]. Polyphenolics can neutralize free radicals by donating an electron or hydrogen atom due to their highly conjugated system and hydroxylation patterns, and reduce the rate of oxidation by suppressing the generation of free radicals, or by deactivation of the reactive species and precursors of free radicals [33]. Besides, some amino acids, such as lysine, histidine, and tyrosine, have been attested to be capable of donating protons to electron-deficient radicals [5,34]. Therefore, the sharp reduction in antioxidant activities of PPP after in vitro digestion might be associated with the decrease of uronic acids and polyphenolics. Besides, it might also be related to the changes of protein to some extent. For instance, after in vitro digestion, the content of Tyr remarkably decreased from 7.30% to 3.11%.

#### 3.2.2. Stability of Antiglycation Activity of PPP after In Vitro Digestion

**Figure 4:**
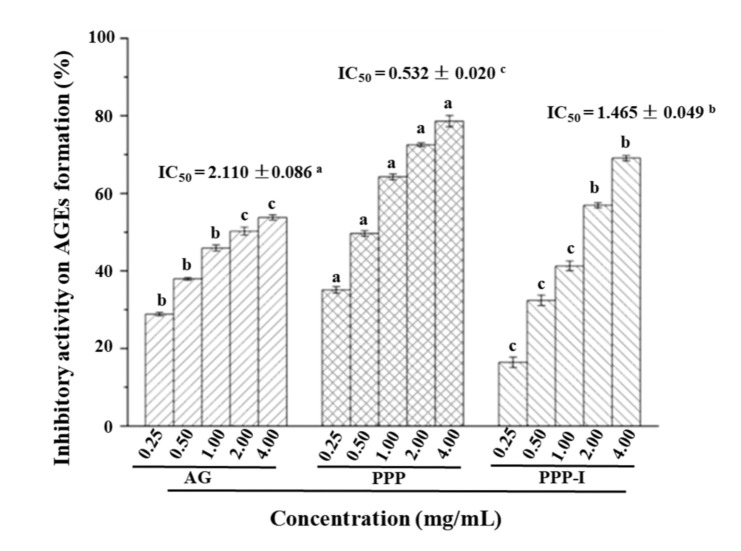
Stability of antiglycation activity of PPP after in vitro digestion. **PPP**, polyphenolic-protein-polysaccharide ternary complexes from *Hovenia dulcis*; **PPP-I**, indicated PPP after in vitro digestion; **AG**, aminoguanidine; The error bars are standard deviations; Significant (*p* < 0.05) differences among AG, PPP, and PPP-I are shown by data bearing different letters (**a**–**c**). Statistical significances (*p* < 0.05) were carried out by one-way analysis of variance (ANOVA) followed by a Duncan’s test.

Glycosylation is a spontaneous non-enzymatic amino-carbonyl reaction between reducing sugars and proteins, and then forms advanced glycation end products (AGEs) which can lead to aging and arteriosclerosis [35]. Therefore, the inhibition of AGEs formation of natural products has attracted considerable attention. As shown in Figure 4, both PPP and PPP-I possessed strong inhibitory effects against the formation of AGEs when compared with the positive control. The IC_50_ values of inhibitory activities against the formation of AGEs of PPP and PPP-I were determined to be 0.532 mg/mL and 1.465 mg/mL, respectively, indicating that the inhibitory effect of PPP against the formation of AGEs significantly decreased after in vitro digestion. Former reports have proven that the oxidation is the main reason for the formation of AGEs, and the high antiglycation activity may be associated with its strong antioxidant activity and high content of polyphenolics [36]. Therefore, the significant decrease in antiglycation activity of PPP after in vitro digestion might be related to the reduction of its antioxidant activities and the decrease of its total polyphenolics.

#### 3.2.3. Stability of Inhibitory Activity against α-Glucosidase of PPP after In Vitro Digestion

**Figure 5:**
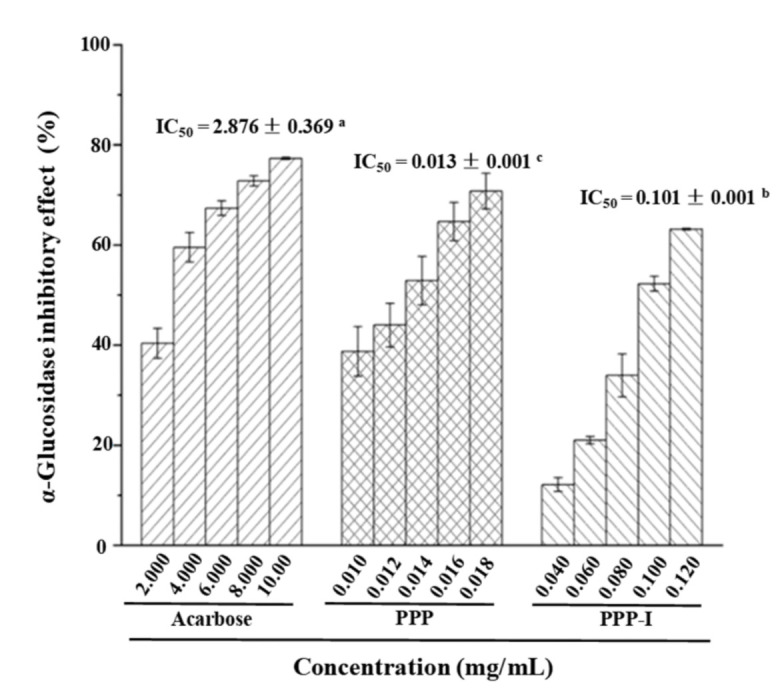
Stability of inhibitory effect on α-glucosidase of PPP after in vitro digestion. **PPP**, polyphenolic-protein-polysaccharide ternary complexes from *Hovenia dulcis*; **PPP-I**, indicated PPP after in vitro digestion; The error bars are standard deviations; Different superscript lowercase letters (**a**–**c**) indicated significance (*p* < 0.05). Statistical significances (*p* < 0.05) were carried out by one-way analysis of variance (ANOVA) followed by a Duncan’s test.

As an important enzyme for carbohydrate digestion, α-glycosidase inhibitor can resist metabolic changes related to hyperglycemia and type 2 diabetes on account delaying the release of glucose and control the postprandial hyperglycemia [37]. Previous studies have found that *H. dulcis* exhibits strong in vitro anti-diabetic effects [6]. Hence, the influence of in vitro digestion on the α-glucosidase inhibitory effect of PPP was investigated. As shown in Figure 5, the IC_50_ values of α-glucosidase inhibitory effect of PPP and PPP-I were 0.013 mg/mL and 0.101 mg/mL, respectively, suggesting that both PPP and PPP-I exerted remarkable inhibitory effects against α-glucosidase compared with the positive control (acarbose, IC_50_ = 2.876 mg/mL). However, after in vitro digestion, the α-glucosidase inhibitory effect of PPP obviously decreased. A previous study showed that flavonoids extracted from *H. dulcis* exhibited strong α-glycosidase inhibitory effect [6]. Meanwhile, the high contents of uronic acids and total polyphenolics of PPP could contribute to its strong α-glucosidase inhibitory effect [5]. In addition, the high molecular weight might be also related to the remarkable inhibitory effect of polysaccharide on α-glucosidase [32,38]. Therefore, the reduction in α-glucosidase inhibitory effect of PPP after in vitro digestion might be associated with the decrease of its molecular weight and total polyphenolics.

## 4. Conclusions

In the present study, the in vitro digestion on physicochemical and biological properties of polyphenolic-protein-polysaccharide ternary complexes from *H. dulcis* was investigated. Physicochemical properties of PPP, including contents of reducing sugar release, bound polyphenolics, proteins, and uronic acids, and molecular weight distribution, as well as ratios of monosaccharides and amino acids, obviously changed after in vitro digestion. Meanwhile, the antioxidant activities, antiglycation activity, and inhibitory effect on α-glucosidase of PPP notably decreased after in vitro digestion, which might be largely related to the remarkable decrease of bound polyphenolics. Results from this study can contribute to a better understanding of the potential digestive mechanism of PPP extracted from *H. dulcis*, which possess important implications for the development of PPP as functional foods and medicines.

## Figures and Tables

**Table 1:** Changes in reducing sugar content and chemical compositions of PPP after in vitro digestion.

	PPP	PPP-I
Reducing sugar content (mg/mL)		0.27 ± 0.02
Total polysaccharides (%)	43.94 ± 1.52 ^a^	40.33 ± 4.37 ^a^
Total uronic acids (%)	22.41 ± 1.17 ^a^	17.68 ± 0.73 ^b^
Total polyphenolics (mg GAE/g)	281.93 ± 2.36 ^a^	54.89 ± 0.42 ^b^
Total protein (%)	17.62 ± 0.75 ^b^	22.43 ± 0.89 ^a^

**PPP**, polyphenolic-protein-polysaccharide ternary complexes from *Hovenia dulcis*; **PPP-I**, indicated PPP after in vitro digestion; Values represent mean ± standard deviation, and different superscript lowercase letters (a-b) indicated significance (*p* < 0.05) in each row. Statistical significances (*p* < 0.05) were carried out by one-way analysis of variance (ANOVA) followed by a Duncan’s test.

**Table 2:** Changes in molecular weight (*M_w_*), polydispersity (*M_w_/M_n_*), and monosaccharide compositions of PPP after in vitro digestion.

	PPP	PPP-I
*M_w_* × 10^4^ (Da)		
Peak 1	5.97 (±0.59%)	
Peak 2		4.68 (±1.21%)
Peak 3		1.39 (±4.34%)
*M_w_/M_n_*		
Peak 1	1.76 (±1.11%)	
Peak 2		1.50 (±2.29%)
Peak 3		1.07 (±4.59%)
Monosaccharide compositions and molar ratios		
Galactose	1.00	1.00
Galacturonic acid	1.78	1.30
Arabinose	1.12	1.11
Glucose	0.96	0.34
Rhamnose	0.64	0.83
Mannose	0.15	0.11
Glucuronic acid	0.07	0.06
Xylose	0.06	0.06

**PPP**, polyphenolic-protein-polysaccharide ternary complexes from *Hovenia dulcis*; **PPP-I**, indicated PPP after in vitro digestion. The peaks were the same as in Figure 1.

**Table 3:** Amino acid compositions of PPP and PPP-I.

Amino Acids	PPP (%)	PPP-I (%)
Glutamic acid	12.50 ± 0.59 ^a^	13.19 ± 0.55 ^a^
Aspartic acid	9.65 ± 0.44 ^b^	13.28 ± 0.62 ^a^
Proline	8.83 ± 0.41 ^a^	6.68 ± 0.31 ^b^
Leucine	8.30 ± 0.40 ^a^	5.33 ± 0.24 ^b^
Serine	7.61 ± 0.37 ^a^	7.44 ± 0.32 ^a^
Glycine	7.59 ± 0.32 ^b^	9.80 ± 0.41 ^a^
Tyrosine	7.30 ± 0.22 ^a^	3.11 ± 0.12 ^b^
Threonine	6.92 ± 0.33 ^a^	6.98 ± 0.32 ^a^
Isoleucine	6.26 ± 0.21 ^a^	3.90 ± 0.17 ^b^
Alanine	6.08 ± 0.30 ^a^	6.46 ± 0.27 ^a^
Valine	4.73 ± 0.23 ^a^	4.99 ± 0.23 ^a^
Phenylalanine	4.38 ± 0.11 ^a^	4.61 ± 0.13 ^a^
Arginine	3.69 ± 0.17 ^a^	3.11 ± 0.14 ^b^
Lysine	3.49 ± 0.13 ^b^	6.07 ± 0.28 ^a^
Cystine	1.39 ± 0.05 ^a^	0.84 ± 0.03 ^b^
Histidine	1.08 ± 0.04 ^b^	2.71 ± 0.13 ^a^
Methionine	0.20 ± 0.01 ^b^	1.47 ± 0.07 ^a^
Essential amino acids	41.59 ± 2.01 ^a^	36.49 ± 1.76 ^b^
Non-essential amino acids	58.41 ± 1.92 ^b^	63.51 ± 2.14 ^a^

**PPP**, polyphenolic-protein-polysaccharide ternary complexes from *Hovenia dulcis*; **PPP-I**, indicated PPP after in vitro digestion. Values represent mean ± standard deviation, and different superscript lowercase letters (a-b) indicated significance (*p* < 0.05) in each row. Statistical significances (*p* < 0.05) were carried out by one-way analysis of variance (ANOVA) followed by a Duncan’s test.

## Data Availability

Not applicable.
